# The Association of Micro-RNA 21 and Hypertension: A Meta-Analysis

**DOI:** 10.5334/gh.1325

**Published:** 2024-05-01

**Authors:** Caihong Xin, Wei He, Xin Sun, Mianxian Li, Hongli Wang

**Affiliations:** 1 Department of Endocrinology and Metabolism, The Fourth People’s Hospital of Shenyang, Shenyang, P.R. China; 2 Department of Endocrinology and Metabolism, People’s Hospital of Liaoning Province, Shenyang, P.R. China; 3 Department of Endocrinology and Metabolism, The First Affiliated Hospital of Soochow University, Suzhou, P.R. China; 4 Department of Cardiology, The First Affiliated Hospital of Soochow University, Suzhou, P.R. China; 5 Department of Cardiology, The Second Affiliated Hospital of Dalian Medical University, Dalian, P.R. China

**Keywords:** micro-RNA 21, hypertension, meta-analysis

## Abstract

Hypertension is a multifactorial, complex disease with high morbidity and mortality rates. Studies have found that micro-RNA 21 (miR-21) levels are significantly increased in patients with hypertension. However, other studies have reported opposite results. Therefore, the relationship between miR-21 expression and hypertension remains controversial. This meta-analysis was conducted to statistically evaluate the miR-21 levels of patients with hypertension. A literature research was conducted using Web of Science, Embase, PubMed, and CNKI. To search for titles or abstracts, ‘hypertension’ in combination with the terms ‘miR-21,’ ‘microRNA-21,’ or ‘miRNA-21’ were used as keywords. Standardized mean differences (SMD) with corresponding 95% confidence intervals (CIs) were determined from the results of the meta-analysis. In total, 12 articles were included in this meta-analysis, involving 546 cases and 436 controls. The results of the meta-analysis showed that miR-21 levels in patients with hypertension were significantly higher than those in the controls (SMD: 1.22; 95% CI [0.35, 2.09]). This meta-analysis is the first to evaluate miR-21 in patients with hypertension. MiR-21 may be a new target for the prediction and treatment of hypertension. Further high-quality studies are needed to better support the association between miR-21 and hypertension.

## Introduction

Hypertension is a multifactorial complex disease characterized by a continuous increase in systemic arterial blood pressure when whole blood vessels are under high pressure. Globally, it has high morbidity and mortality rates [1]. It is an important cause of premature death and cardiovascular disease and has created a huge disease burden on society [2]. The pathogenesis of hypertension includes the over-activation of the renin-angiotensin-aldosterone system, over-excitation of the sympathetic nervous system, endothelial dysfunction, oxidative stress, and impaired angiogenesis. However, the potential molecular mechanisms underlying hypertension need to be explored further [3,4].

Recent studies have shown that microRNAs (miRNAs) participate in the physiological and pathological processes of various diseases [5]. MiR-21 is one of the most intensively studied miRNAs in recent years and plays an important role in tumors and the cardiovascular system [6]. Furthermore, miR-21 levels are significantly upregulated in hypertensive individuals [7,8], suggesting that miR-21 is a potential treatment for hypertension. However, other studies have found no significant difference in miR-21 levels between patients with hypertension and healthy controls [9,10]. Therefore, the relationship between miR-21 expression and hypertension remains controversial. The aim of this meta-analysis was to systematically evaluate the relationship between peripheral blood miR-21 levels and hypertension, which can provide a theoretical basis for the pathogenesis and treatment of hypertension.

## Methods

### Search

We searched the Web of Science, Embase, PubMed, and CNKI electronic databases. We searched for papers whose title or abstract contained the term “miR-21,” “microRNA-21,” or “miRNA-21”, in combination with the term “hypertension”. We focused our search on the period 1990–2022, and English and Chinese was used. The references of the retrieved articles were checked to ensure that no additional eligible studies were included. No unpublished studies were identified to date. This systematic review and meta-analysis was registered in PROSPERO under CRD42022358397. The supplementary data contains a list of all the items that should be reported for systematic reviews and meta-analyses (S1).

### Inclusion criteria

The studies included in this meta-analysis met the following criteria: (1) they were a case-control or prospective design, (2) they were detailed data about miR-21 levels in patients with hypertension and healthy controls, and (3) their language was English or Chinese.

### Data extraction and risk of bias

As part of the preliminary screening process, two reviewers independently implemented the search strategy and read the papers’ titles and abstracts to exclude studies that did not meet the inclusion criteria. To determine whether the studies met the inclusion criteria, the two reviewers methodologically reviewed the full text. If the author’s information was incomplete, they could contact and crosscheck the author. If the conclusions of the two evaluators were inconsistent, the differences were resolved through discussion. If the discussion failed to resolve any differences, it was judged and arbitrated by a third researcher. The Newcastle-Ottawa Scale (NOS) is a tool for assessing the risk of a bias in observational studies, recommended by the Cochrane Collaboration [11]. The quality of the included studies was evaluated according to the NOS. The NOS evaluates three aspects: the selection method of the case and control groups, the comparability of the case and control groups, and the evaluation method of exposure. The NOS ranged from zero to nine stars, and quality was based on star scores.

### Statistical analysis

We assessed heterogeneity among the included studies using the I^2^ statistic and presented the data as standardized mean differences (SMDs) and 95% confidence intervals (CIs). Fixed-effects models were used if I^2^ was < 50% and heterogeneity among studies was low or moderate; otherwise, random-effects models were used if I^2^ was > 50%. A sensitivity analysis was performed to evaluate the stability of the results. Begg’s and Egger’s tests were used to detect publication bias. *P* < 0.05 was set as the significance level. Data analysis was performed using Stata version 12.0 (College Station, TX).

## Results

**Figure 1:**
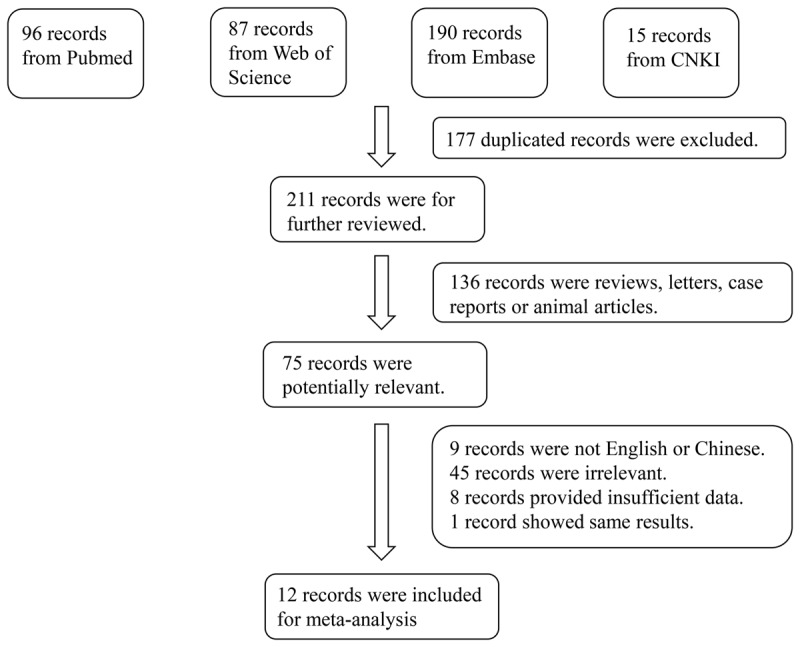
Flowchart of the detailed procedure for the inclusion or exclusion of selected studies.

**Figure 2:**
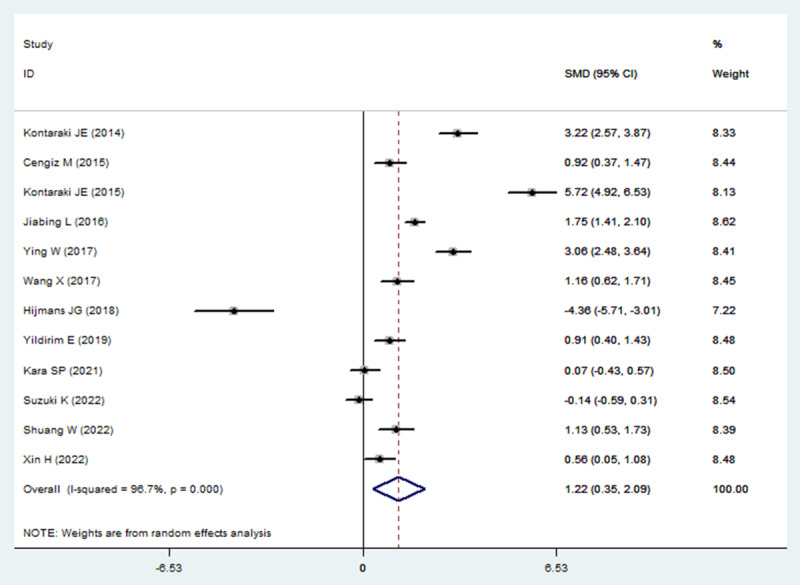
Forest plots of micro-RNA 21 levels in patients with hypertension compared with the controls. Diamond represents standardized mean differences (SMDs) at 95% confidence intervals (CIs).

A total of 388 articles were initially retrieved, and 12 qualified articles were obtained through preliminary screening and quality evaluation [7,8,9,10,12,13,14,15,16,17,18,19]. The publications contained 546 cases and 436 controls. The literature retrieval process is shown in Figure 1, and the baseline data and quality evaluation of the included case-control studies are shown in Table 1. The results of the meta-analysis showed that miR-21 levels were higher in patients with hypertension than in the controls (SMD: 1.22; 95% CI [0.35, 2.09]). The forest plots are shown in Figure 2.

**Table 1:** The characteristic of the selected studies in the meta-analysis.


AUTHOR	YEAR	REGION	AGE(YEARS)		SEX(M/F)		SAMPLE SIZE(N)		MICRORNA 21	
			
			CASE	CONTROL	CASE	CONTROL	CASE	CONTROL	CASE	CONTROL

Kontaraki JE	2014	Greece	60.42 ± 9.6	56.69 ± 8.59	29/31	13/16	60	29	3.08 ± 0.32	2.06 ± 0.31

Cengiz M	2015	Turkey	47.3 ± 5.6	45.4 ± 5.3	15/13	18/10	28	28	84.40 ± 92.30	24.10 ± 5.30

Kontaraki JE	2015	Greece	62.51 ± 9.7	58.8 ± 8.3	50/52	14/16	102	30	2.75 ± 0.15	1.82 ± 0.20

Jiabing L	2016	China	–	–	90/0	90/0	90	90	7.87 ± 5.46	1.03 ± 0.80

Ying W	2017	China	55.28 ± 8.03	53.20 ± 5.71	26/24	25/25	50	50	33.00 ± 3.01	26.14 ± 1.03

Wang X	2017	China	48.2 ± 12.8	46.5 ± 10.3	38/29	10/40	55	20	1.29 ± 0.52	0.72 ± 0.39

Hijmans JG	2018	America	59 ± 2	56 ± 1	10/5	10/5	15	15	1.32 ± 0.25	2.50 ± 0.29

Yildirim E	2019	Turkey	46.5 ± 5.9	44.7 ± 5.7	19/13	20/12	32	32	81.90 ± 88.90	22.40 ± 23.80

Kara SP	2021	Turkey	52.67 ± 7.49	48.84 ± 9.35	9/21	9/23	30	32	3.20 ± 10.50	2.60 ± 5.50

Suzuki K	2022	Japan	62.3 ± 5.9	59.6 ± 9.7	13/16	20/35	29	55	0.89 ± 1.01	1.11 ± 1.73

Shuang W	2022	China	51.66 ± 7.84	50.84 ± 7.47	15/10	12/13	25	25	2.25 ± 0.87	1.44 ± 0.52

Xin H	2022	China	49.40 ± 10.27	41.86 ± 10.10	39/61	52/48	30	30	1.42 ± 1.18	0.88 ± 0.66


### Sensitivity analysis and publication bias

**Figure 3:**
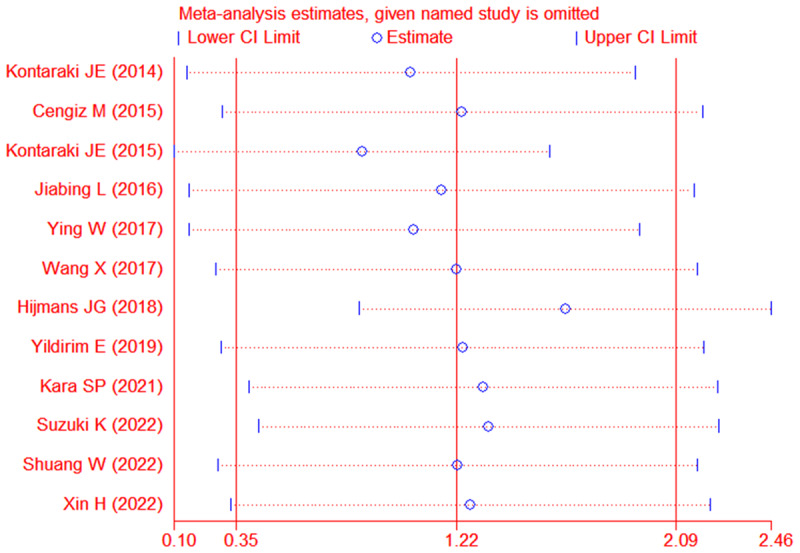
The sensitivity analysis results of micro-RNA 21 levels in patients with hypertension compared with the controls.

Each study was subjected to a sensitivity analysis to determine its influence. Sensitivity analysis showed no significant differences from our previous estimates, indicating that a single study had a marginal impact on the overall estimate (Figure 3). Accordingly, the meta-analysis yielded stable results. A thorough and comprehensive search of the databases was conducted. Begg’s and Egger’s tests were conducted to identify whether publication bias was present in the reviewed studies. The results (*P* > 0.05) indicated that there was no publication bias.

## Discussion

Among more than 2,000 microRNAs, miR-21 is highly expressed in human hearts and has earned the interest of researchers as a potential biomarker in a wide range of common heart conditions [20]. This systematic review is the first to evaluate the association between miR-21 and hypertension. In this meta-analysis, we concluded that the expression of miR-21 in patients with hypertension is significantly higher than that in healthy controls (SMD: 1.22; 95% CI [0.35, 2.09]). MiR-21 is a non-coding RNA with a length of 22 nucleotides and is homologous in humans, rats, and mice [20]. Human miR-21 is located on chromosome 17q23.2. Although it overlaps with transmembrane protein 49 (TMEM-49), it is not regulated by the TMEM-49 promoter and is independently transcribed in its own relatively conservative promoter. At the post-transcriptional level, it participates in the pathological process of hypertension by regulating a variety of target genes related to hypertension [21]. MiR-21 is widely expressed in mammalian tissues, organs, cells, and the blood. Owing to its stability, pathological specificity, and sensitivity in the circulatory system, it may be a potential biomarker for the diagnosis, treatment, and prognosis of hypertension [22,23]. The lack of miR-21 in macrophages can promote cell apoptosis, plaque necrosis, and vascular inflammation during atherosclerosis formation [24]. Anti-miR-21 therapy has a good fibrotic inhibition effect in large-animal models of heart failure and can improve heart function [25].

MiR-21 expression was positively correlated with blood pressure in spontaneous hypertension rats (SHR) and hypertensive patients. miR-21 expression was increased in SHR compared to Wistar rats, and circulating miR-21 levels in hypertensive patients were similarly higher than in controls [26]. Overexpression of miR-21 by adeno-associated virus was sufficient to decrease blood pressure and alleviate cardiac hypertrophy in SHRs by upregulating mitochondrial translocation. Nuclear DNA synthesis was increased, and mitochondrial DNA synthesis was decreased, causing an increase in intracellular ROS. The authors concluded that induced miR-21 was part of the compensatory program [27]. Animal studies have found that the level of miR-21 in the skeletal muscle of hypertensive rats is higher than that in normotensive rats. Lowering blood pressure can also decrease the expression of miR-21 [28]. In another study, researchers found a positive correlation between miR-21 expression and hypertension. Moreover, they observed that the expression of mtDNA-encoded cytochrome b (mt-Cytb) decreased in SHRs. This seemed to cause an increase in the mitochondrial reactive oxygen species (mt-ROS). In addition, recombinant adeno-associated virus–mediated delivery of miR-21 was sufficient to reduce blood pressure and attenuate cardiac hypertrophy in SHRs [29]. Wang *et al*. showed that intravenous delivery of recombinant adeno-associated virus-mediated miR-21–3p expression induced a persistent attenuation of hypertension, with marked amelioration of target organ damage, including cardiac hypertrophy and fibrosis, and artery and kidney fibrosis in SHRs, whereas miR-21–3p tough decoys counteracted these effects. Computational prediction coupled with biochemical experiments revealed that the miR-21–3p mediated hypotensive reduction effect was accomplished by regulating the phenotypic switch of vascular smooth muscle cells *via* suppression of the adrenal α2B-adrenergic receptor in arteries [30].

Most investigations highlighted miR-21 cardioprotective functions in heart injury, while some other studies showed that this miR-21 is elevated in the serum of patients, promoting fibrosis and cardiac dysfunction [23,25,27,29]. This dual role can be explained by the fact that miR-21 has multiple regulatory functions depending on the microenvironment, downstream signaling, and target genes, which indicates that cell-type-specific investigations should receive more attention. With further investigations, miR-21 could be considered a novel tailored therapy with favorable outcomes [31].

This meta-analysis aimed to statistically evaluate the association between miR-21 levels and hypertension. However, this study had some limitations. Due to the lack of large sample studies, most studies included in this meta-analysis were small. Furthermore, the studies included in our meta-analysis were conducted in only five countries. Finally, different detection methods for miR-21 have also been used in these studies. All these factors may have affected the results; therefore, the results of this meta-analysis should be interpreted cautiously. More high-quality, large-scale studies in different countries are required.

## Conclusion

This systematic review was the first to evaluate the association between miR-21 and hypertension. Our findings suggest that the expression of miR-21 in patients with hypertension is significantly higher than that in healthy controls. MiR-21 could be a new target for the prediction and treatment of hypertension. Further high-quality studies are needed to better support the association between miR-21 and hypertension.

## Data Accessibility Statement

All data relevant to the study are included in the article or uploaded as supplemental information.

## Additional File

The additional file for this article can be found as follows:

10.5334/gh.1325.s1 Supplementary File. S1 Preferred reporting items for systematic review and meta-analyses (PRISMA) checklist.
